# Screening *Arabidopsis thaliana* mutants for low sensitivity to manganese identifies novel alleles of *NRAMP1* and *PGSIP6*

**DOI:** 10.1093/jxb/ery018

**Published:** 2018-01-20

**Authors:** Bian Bian, Sae Kageshima, Kenji Yano, Toru Fujiwara, Takehiro Kamiya

**Affiliations:** 1 Department of Applied Biological Chemistry, Graduate School of Agricultural and Life Sciences, The University of Tokyo, Yayoi, Bunkyo-ku, Tokyo, Japan; 2 Precursory Research for Embryonic Science and Technology (PRESTO), Japan Science and Technology Agency, Saitama, Japan

**Keywords:** *Arabidopsis thaliana*, Golgi, manganese deficiency, NRAMP1, PGSIP6, screening

## Abstract

Manganese (Mn) is an essential micronutrient; however, few genes required for growth under low-Mn conditions have been identified. In this study, we isolated *Arabidopsis thaliana* mutants sensitive to low-Mn conditions from ethyl methanesulfonate-mutagenized seeds. Among them, we identified the causal genes of two mutants. One mutant (35-34) exhibited a short root phenotype and low Mn concentration in the shoots. The other mutant (30-11) exhibited a small shoot phenotype with Mn concentrations similar to the control. Genetic mapping, allelism tests, and gene complementation tests identified the causal genes as *At1g80830* (*NRAMP1*) for 35-34 and *At5g18480* (*PGSIP6*) for 30-11. NRAMP1 was previously reported to be essential for Mn uptake under low-Mn conditions, thus validating our screening method. *PGSIP6* encodes inositol phosphorylceramide glucuronosyltransferase, which is involved in glycosyl inositol phosphorylceramide sphingolipid glycosylation. PGSIP6-green fluorescent protein was localized to the Golgi apparatus, which is consistent with its function in the glycosylation of sphingolipids. Our screening identified a novel gene required for low-Mn tolerance, and we also provide new insights towards understanding the physiological function of PGSIP6.

## Teaser

Screening Arabidopsis mutants for sensitivity to low-Mn conditions identifies novel alleles for NRAMP1, a previously known Mn transporter, and PGSIP6, a phosphorylceramide glucuronosyltransferase.

## Introduction

Manganese (Mn) is an essential micronutrient for all known organisms, and is the second most abundant transition element constituting the Earth’s crust (Yang *et al.*, 2008). The dominant form of Mn in aerated soils is the tetravalent state, but only the form Mn (Mn^2+^) is the available for uptake by plants. Therefore, although soil contains a large amount of Mn, the quantity that can be used by organisms is quite low (Kabata-Pendias, 2011).

Mn deficiency a physiological disorder that is widely observed in agriculture. Mn deficiency often occurs in alkaline or calcareous soils due to the low bioavailability and immobilization of Mn^2+^ (Marschner, 1995). Typical Mn-deficiency symptoms include diffused interveinal chlorosis in younger leaves due to the low phloem mobility of Mn^2+^ from old to young leaves (Marschner, 1995; Schmidt *et al.*, 2016). In addition, it has been reported that some species become more susceptible to cold stress and pathogen infection under Mn deficiency (Marschner, 1995). Therefore, it is important to have a better understanding of the adaptation mechanisms to low-Mn conditions.

Mn acting as a component of manganese superoxide dismutase (MnSOD), an important antioxidant defense in mitochondria, exists in nearly all living cells exposed to oxygen (Socha and Guerinot, 2014). Mn is also required as a catalyst in the oxygen-evolving complex (OEC) of photosystem II, which is responsible for water photolysis using sunlight and is present in the thylakoids of plants, algae, and cyanobacteria (Kern *et al.*, 2005; Umena *et al.*, 2011; Schmidt *et al.*, 2016). Mn functions as a co-factor or activator for a wide variety of catalysts in decarboxylation, oxidation–reduction, and hydrolytic reactions (Marschner, 1995).

Mn is also required for glycosylation. ß1,3-galactosyltransferase1 (GALT1) catalyses the transfer of ß1,3-linked galactose residues onto N-glycans (Strasser *et al.*, 2007). α1,3-fucosyltransferases catalyses the addition of L-fucose from GDP-Fuc to the reducing terminal N-acetylglucosamine (GlcNAc) residue of N-glycans (Both *et al.*, 2011). GlcA substitution of xylen 1 (GUX1) catalyses the addition of glucuronosyl substitutions on the xylan backbone (Rennie *et al.*, 2012). All of these enzymes are localized to the Golgi apparatus and require Mn^2+^ for their activity.

Because Mn plays such an important role in physiological activities, plants are capable of maintaining their cellular Mn concentration within a certain range. Several genes have been reported to regulate Mn distribution in plants. The natural resistance-associated macrophage protein (NRAMP) family are transporters responsible for Mn distribution and exist in a wide variety of species, ranging from bacteria to humans (Cellier *et al.*, 1995; Nevo and Nelson, 2006). There are six NRAMP family members in *Arabidopsis thaliana*. AtNRAMP1 is localized to the plasma membrane as well as to intracellular membranes including the Golgi apparatus (Cailliatte *et al.*, 2010; Agorio *et al.*, 2017). NRAMP1 is involved in Mn absorption from the soil and is required for the growth under low-Mn conditions (Cailliatte *et al.*, 2010). AtNRAMP2 is localized on the trans-Golgi network and is required for root growth under low-Mn conditions (Gao *et al.*, 2018). AtNRAMP3 and AtNRAMP4 are localized to the vacuolar membrane and have important roles in Mn allocation to the chloroplasts (Lanquar *et al.*, 2010). A double-mutant of *AtNRAMP3* and *AtNRAMP4* shows impaired growth due to the reduced function of photosystem II under low-Mn conditions (Lanquar *et al.*, 2010). In rice, OsNRAMP5 localized in the plasma membrane contributes to Mn uptake (Ishimaru *et al.*, 2012).

In addition to the NRAMP family, several other Mn transporters have been identified in plants. AtECA3 belongs to the Ca^2+^-ATPase subfamily, which has also been reported to be involved in Mn transport. It has been shown that AtECA3 is localized to the Golgi apparatus and mutants where its function is disrupted show poor growth under low-Mn conditions (Mills *et al.*, 2008). Iron-regulated transporter 1 (IRT1) is a metal transporter that acts on both Fe and Mn (Vert *et al.*, 2002). Transcripts of *HvIRT1* in barley are induced under low-Mn and low-Fe conditions. Since Mn^2+^ absorption increases in proportion to the expression of *HvIRT1*, it is thought that it promotes efficient absorption of Mn (Pedas *et al.*, 2008). Arabidopsis metal tolerance protein 11 (AtMTP11), belonging to the cation diffusion facilitator (CDF) family, is localized to pre-vacuolar compartments and confers resistance to excess Mn (Delhaize *et al.*, 2007).

In this study, we isolated mutants sensitive to low-Mn conditions and identified the causal genes. One was a previously known gene, *NRAMP1*, and the other was *PGSIP6*, which encodes an enzyme required for the formation of glycosyl inositol phosphorylceramide (GIPC) sphingolipids (Tartaglio *et al.*, 2017). Our study provides new insights connecting GIPC sphingolipid synthesis with a mechanism for low-Mn tolerance in plants.

## Material and methods

### Plant material and cultivation conditions

*Arabidopsis thaliana* (L.) Heynh was used as the plant material with Columbia-0 (Col-0) used as the control. Ethyl methanesulfonate (EMS)-mutagenized seeds were obtained from Lehle Seeds (http://www.arabidopsis.com/). T-DNA insertion mutants were obtained from the Arabidopsis BioResource Center (ABRC; http://abrc.osu.edu/) or the Nottingham Arabidopsis Stock Centre (NASC; http://arabidopsis.info/).

Plants were grown on MGRL medium (Fujiwara *et al.*, 1992). To adjust the Mn concentration in the medium, Mn hexahydrate (MnSO_4_.6H_2_O) was supplied.

Plant cultivation was conducted as follows. Seeds were soaked in a 10% sodium hypochlorite solution for 5 min and washed five times with sterilized ultrapure water. The sterilized seeds were sown in the MGRL medium and subjected to a vernalization treatment at 4 °C for 1–3 d. The plates were placed in a growth chamber (LPH-350S, Nippon Medical Equipment Co., Ltd.) under long-day conditions (16/8 h light/dark cycle) at 22 °C. After 2 weeks, plants were harvested for analysis.

### Screening for mutants sensitive to low-Mn conditions

First, about 15 000 EMS-mutagenized M_2_ seeds derived from 15 000 M_1_ seeds were sown onto MGRL medium solidified with 1.2% agar (Type A; Sigma-Aldrich) without Mn supplementation (0 μM Mn). The 0 µM Mn agar plate was calculated to contain 0.47 µM Mn according to Jain *et al.* (2009). The seeds were grown for 10 d (25 seeds per 140 cm^2^) and plants with poor growth compared to the wild-type were selected and transplanted to normal Mn conditions (10 μM Mn). At 7 d after transplanting, plants whose growth recovered were selected as mutants. Secondary screening was performed using M_3_ seeds derived from M_2_. The seeds were sown in the low- and normal-Mn media (8 seeds per 140 cm^2^) and after 10–14 d the mutants that showed growth inhibition only in low-Mn conditions were selected as low-Mn-sensitive mutants.

### Determination of Mn concentrations

Plants were grown for 14 d under different Mn concentrations. The shoots and roots of 5–10 plants were harvested and pooled for each sample, rinsed twice with ultrapure water, and then dried at 60 °C for 48 h. The weights of the dried samples were measured with an electronic balance, and samples were then transferred to Teflon tubes for nitric-acid digestion. Nitric acid (1 ml) was added to each sample (dry weight 1–5 mg) and digestion took place at 100 °C. After digestion, the samples were dissolved in 2 ml of 0.08 N nitric acid containing 1 ppb indium as an internal standard. Element concentrations were then measured using inductively coupled plasma mass spectrometry (ICP-MS: model SPQ 9700; SII Nano Technology, Seiko).

### Map-based cloning

For genetic mapping, mutants 35-34 and 30-11 were crossed with the Landsberg *erecta* (L*er*) accession, and the respective self-fertilized F_2_ populations were used for mapping. The F_2_ plants were grown in media without Mn, after which genomic DNA was prepared from those plants showing the mutant phenotype. Simple sequence length polymorphism markers were used for the mapping.

### Genome sequencing of mutants by SOLiD^TM^ next-generation sequencing

For mutant 30-11, after rough mapping, a next-generation sequencer system (SOLiD™, Applied Biosystems) was used to identify the mutation in the mapped region, as described previously (Tabata *et al.*, 2013). Genomic DNA extraction was conducted using DNeasy (Qiagen, MD, USA), and sample sequence analysis was performed using the SOLiD system at the University of Tokyo’s Graduate School of Frontier Sciences Omics Information Center (http://www.k.u-tokyo.ac.jp/omics/). The results of the analysis were visualized using IGV (Integrative Genome Viewer, Broad Institute; http://software.broadinstitute.org/software/igv/).

### RNA isolation, reverse transcription, and quantitative RT-PCR

Plants were cultivated for 10 d under either 0 or 10 μM Mn and were then dissected into shoots and roots. The tissues were disrupted twice at 2000 rpm for 15 s using a multi-bead shocker [MB 755 U (S) type, Yasui Kikai Co., Ltd.]. Total RNA was extracted using NucleoSpin^®^ RNA Plant with rDNase (Macherey-Nagel GmbH & Co. KG).

The extracted RNA was reverse-transcribed into cDNA using a Prime Script RT-PCR Kit (Takara, Japan). The diluted cDNA was applied for quantitative PCR using SYBR®PremixEx TaqII (Takara). Transcripts were detected using the following primers: for *NRAMP1*, 5′-ATTCATTTCCAGTTCCGGCG-3′ and 5′-CCTGGGTCTGGCTTTGAGTA-3′; For *PGSIP6*, 5′-CCCA CCATTAGACCCAACCT-3′ and 5′-AGGGATGGCTTTTCT GGCTA-3′.

### Plasmid construction and plant transformation

For the complementation test, a genomic fragment containing 2.3 kbp upstream of the first codon of *PGSIP6* was amplified by PCR using PrimeSTAR® Max DNA Polymerase (Takara) with primers (5′-CACCCATTGGCACCTGTTGTTGTC-3′ and 5′-ACAGAGGAAACATAGGGAATTTG-3′). PCR products were cloned into pENTR™/D-TOPO® (Life Technologies) and then transferred to a Gateway destination vector pMDC107 (Curtis and Grossniklaus, 2003) using LR clonase. The obtained plasmids were transformed into *Agrobacterium tumefaciens* GV 3101::pMP90 and then transformed into mutant 30-11 using the floral dip method (Clough and Bent, 1998).

### Subcellular observations of PGSIP6

Plants grown under normal Mn conditions for one week were observed using a confocal laser scanning microscope (FV1000, Olympus). The roots were stained with propidium iodide (PI, 10 µg ml^–1^). For co-localization of the Golgi marker protein ST-mRFP (Ito *et al.*, 2012), F_1_ plants ST-mRFP/Col-0 (red fluorescent protein) and PGSIP6-GFP/30-11 (green fluorescent protein) were used. GFP, PI, and mRFP fluorescence were observed with the following excitation and emission settings: 488 nm and 505–540 nm band pass filter for GFP, and 559 nm and 570–670 nm for PI and mRFP.

## Results

### Isolation of mutants 35-34 and 30-11

We screened for low-Mn-sensitive mutants using EMS-mutagenized Arabidopsis seeds, selecting plants with halted or reduced growth under 0 µM Mn compared to growth under normal Mn conditions (10 µM). Through this screening, we obtained eight mutant lines (Supplementary Fig. S1 at *JXB* online) and from among these, we focused on two mutants, 35-34 and 30-11.

**Fig. 1.**
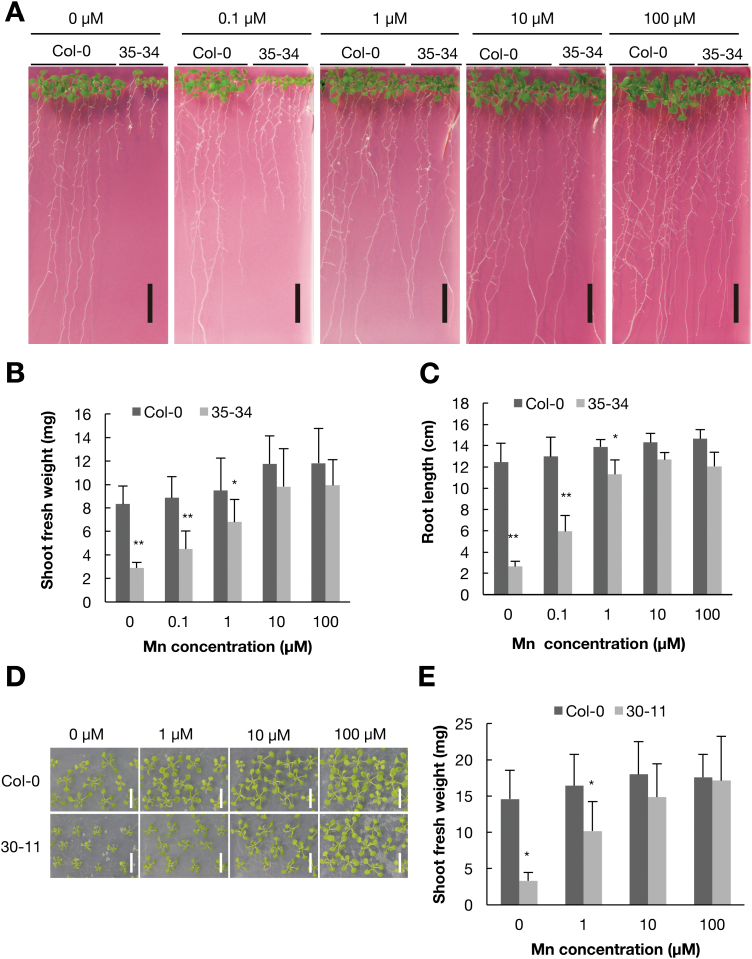
Low-manganese sensitivity of mutants 35-34 and 30-11. (A) The phenotypes of wild-type Col-0 and mutant 35-34 under various Mn concentrations. Plants were grown on MGRL medium at 22 °C for 14 d. Scale bars indicate 1 cm. (B) Fresh weight of the shoots of Col-0 and mutant 35-34 under different Mn concentrations. (C) The length of the main root of Col-0 and mutant 35-34. (D) The phenotype of Col-0 and mutant 30-11 under various Mn concentrations. Plants were grown on MGRL medium for 14 d. Scale bars indicate 1 cm. (E) Shoot fresh weight of Col-0 and mutant 30-11. Plants were grown under various Mn concentrations for 14 d. Data in (B, C, E) are means (+SD), *n*=5–8 (B, C), *n*=9–12 (E). Asterisks indicate significant differences between Col-0 and the mutants as determined by Student’s *t*-test (*P<0.05, **P<0.01).

One of the isolated lines, 35-34, showed a short root and small shoot phenotype under 0 μM Mn (Fig. 1A), with the shoot fresh weight being lower than that in the wild-type under low-Mn conditions (0, 0.1, and 1 µM; Fig. 1B). Under normal and high-Mn (100 µM) conditions, the shoot weight was similar to that of the wild-type. The root length of mutant 35-34 showed similar tendencies as observed for shoot weight (Fig. 1C).

Another mutant line, 30-11, showed a small shoot phenotype under low-Mn conditions (Fig. 1D), with the shoot fresh weight being was less than that of Col-0 under 0 and 1 µM Mn (Fig. 1E). Under normal and high-Mn conditions, the shoot fresh weight of mutant 30-11 and the wild-type were comparable. In contrast to 35-34, the root length of mutant 30-11 was comparable to that of the wild-type under both normal and Mn-deficient conditions (data not shown). These results suggested that the causal gene of mutant 30-11 confers low-Mn tolerance to shoots.

**Fig. 2.**
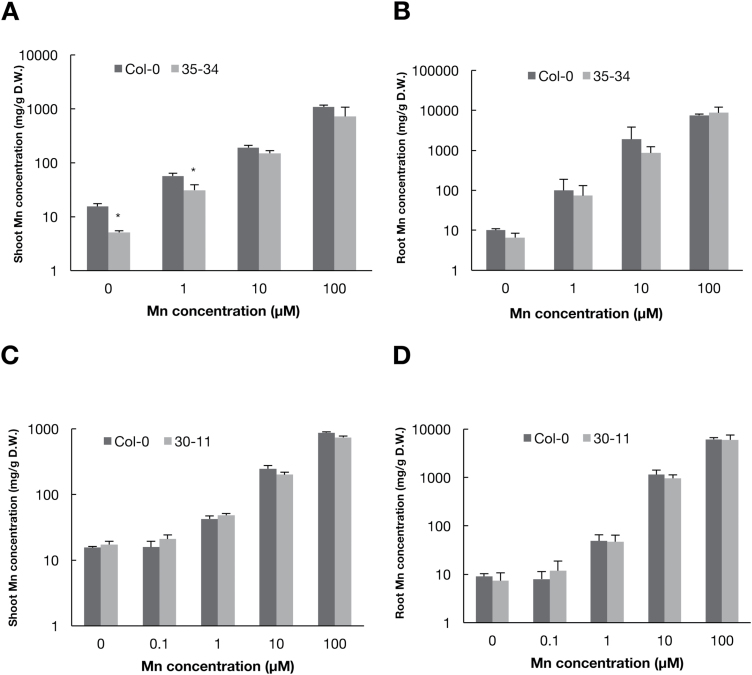
Mn concentrations of wild-type Col-0 and mutants 34–35 and 30-11. Plants were grown for 14 d under various concentrations of Mn. Mutant 35-34 was grown horizontally and mutant 30-11 was grown vertically. Mn concentrations in shoots and roots were determined using ICP-MS and are expressed as means (+SD). Mn concentrations in (A) shoots and (B) roots of mutant 34–35 (*n*=8). Mn concentrations in (C) shoots and (D) roots of mutant 30-11 (*n*=8–14). Asterisks indicate significant differences between Col-0 and the mutants as determined by Student’s *t*-test (*P<0.05).

To observe the function of the causal gene, we determined the Mn concentration in the mutants, using inductively coupled plasma mass spectrometry (ICP-MS) after growing plants under various Mn conditions. Under low-Mn conditions (0 and 1 μM), the shoots of 35-34 had lower Mn concentrations than those of Col-0 (Fig. 2A); however, no significant differences were observed for root concentrations (Fig. 2B). This suggested that the causal gene of 35-34 is responsible for Mn transport. The Mn concentrations in shoots and roots of 30-11 were also determined using ICP-MS, and no significant differences were found between the mutant and Col-0, irrespective of Mn concentration in the medium (Fig. 2C, D), suggesting that the causal gene of 30-11 would not be involved in Mn transport.

### Identification of the causal gene of mutant 35-34

**Fig. 3.**
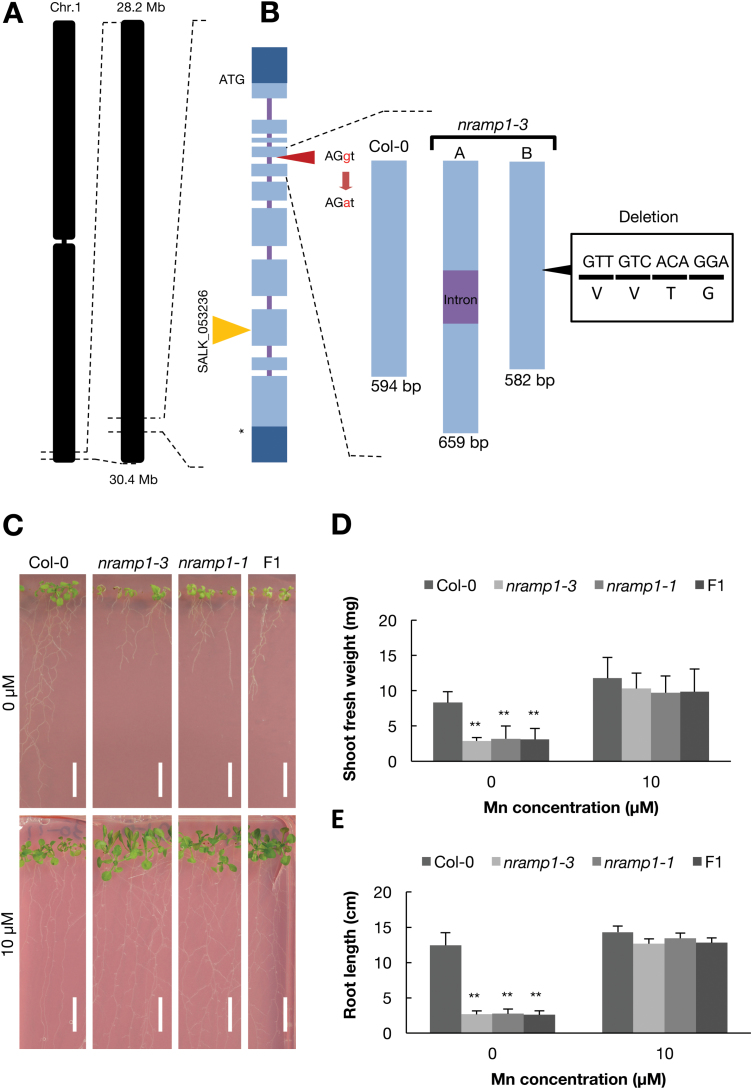
Identification of *NRAMP1* as the causal gene. (A) Genetic mapping and the mutation site of *nramp1-3*, the exon–intron structures of the *NRAMP1* gene, and the insertion site of T-DNA in SALK_053236 (*nramp1-1*). (B) A schematic diagram of the *NRAMP1* gene in the wild-type and the splicing junction mutation in *nramp1-3*. Light blue boxes indicate exons encoding a protein, dark blue boxes are untranslated regions, and the bars between them are introns. ‘A’ represents the intron-inserted transcript and indicates the upper band shown in Supplementary Fig. S2A. ‘B’ represents the exon transcript that has a 12-bp deletion and indicates the bottom band in Supplementary Fig. S2A. (C) Phenotype of wild-type Col-0, *nramp1-3*, *nramp1-1*, and F_1_ plants between *nramp1-3* and *nramp1-1* under Mn deficiency and normal conditions. Plants were grown for 14 d. Scale bars indicate 1 cm. Fresh weight of shoots (D) and root length (E) of Col-0, *nramp1-3*, *nramp1-1*, and F_1_ plants. Data are means (+SD), *n*=6. Asterisks indicate significant differences between Col-0 and the mutants as determined by a Tukey–Kramer test (**P<0.01).

To identify the causal gene of 35-34, we crossed the mutant with Landsberg *erecta* (L*er*) and obtained a selfed F_2_ population. The segregation ratio of the F_2_ plants was 241:85 wild-type to mutant phenotype. According to a χ^2^ test, the segregation ratio was not significantly different from the expected ratio of 3:1 (*P*=0.65), suggesting that the phenotype of mutant 35-34 is caused by a single recessive mutation. Genetic mapping revealed that the causal gene of the mutant was located between 28.16 and 30.42 Mb on chromosome 1 (Fig. 3A). Based on the TAIR10 database (https://arabidopsis.org), there are 836 genes within this region. Among these, we found a gene encoding a Mn^2+^ transporter, *NRAMP1*. It has been reported that the disruption of *NRAMP1* makes plants sensitive to low-Mn conditions: shoots are small and etiolated and the root length is short compared with that of the wild-type (Cailliatte *et al.*, 2010).

We then determined the genomic sequence of *NRAMP1* of mutant 35-34 using Sanger sequencing and found a mutation. Therefore, and hereafter, we refer to 35-34 as *nramp1-3*. The mutation is a nucleotide substitution from a guanine (G) to an adenine (A) at the acceptor site of the splicing junction between the fourth exon and the fourth intron (Fig. 3B). To examine the effects of the mutation in *NRAMP1* splicing, we performed PCR using *nramp1-3* cDNA as a template. Under both low- and normal Mn conditions, mis-spliced *NRAMP1* fragments were present in both shoots and roots (Supplementary Fig. S2A). We then determined the sequences of the mis-spliced fragments and found retention of the fourth intron (*nramp1-3a*) and a 12-bp deletion [a four-amino-acid (VVTG) deletion] in the fourth exon (*nramp1-3b*). The intron retention leads to an early stop-codon 51 nucleotides downstream from the fourth exon–intron junction and the addition of 17 amino acids (Supplementary Fig. S2B).

To confirm that the mutation in *NRAMP1* was responsible for the low-Mn-sensitivity phenotype of *nramp1-3*, we obtained a T-DNA insertion line of *NRAMP1* (SALK_053236: *nramp1-1*) (Fig. 3B). When grown under low Mn, *nramp1-3* and *nramp1-1* showed 2.5-fold less shoot fresh weight and shorter root length than the wild-type; However, no differences were found between the wild-type and mutant plants in the presence of normal Mn (10 μM) (Fig. 3C–E). In addition, we performed an allelism test between the *nramp1-3* and *nramp1-1* lines. The phenotype of the F_1_ crosses between the *nramp1-3* and *nramp1-1* lines was similar to the mutants under low-Mn conditions (0 μM) (Fig. 3C–E). These findings indicate that the causal gene of *nramp1-3* is *NRAMP1*, and hence through this study we obtained a novel *nramp1* mutant allele.

### Identification of the causal gene of 30-11

**Fig. 4.**
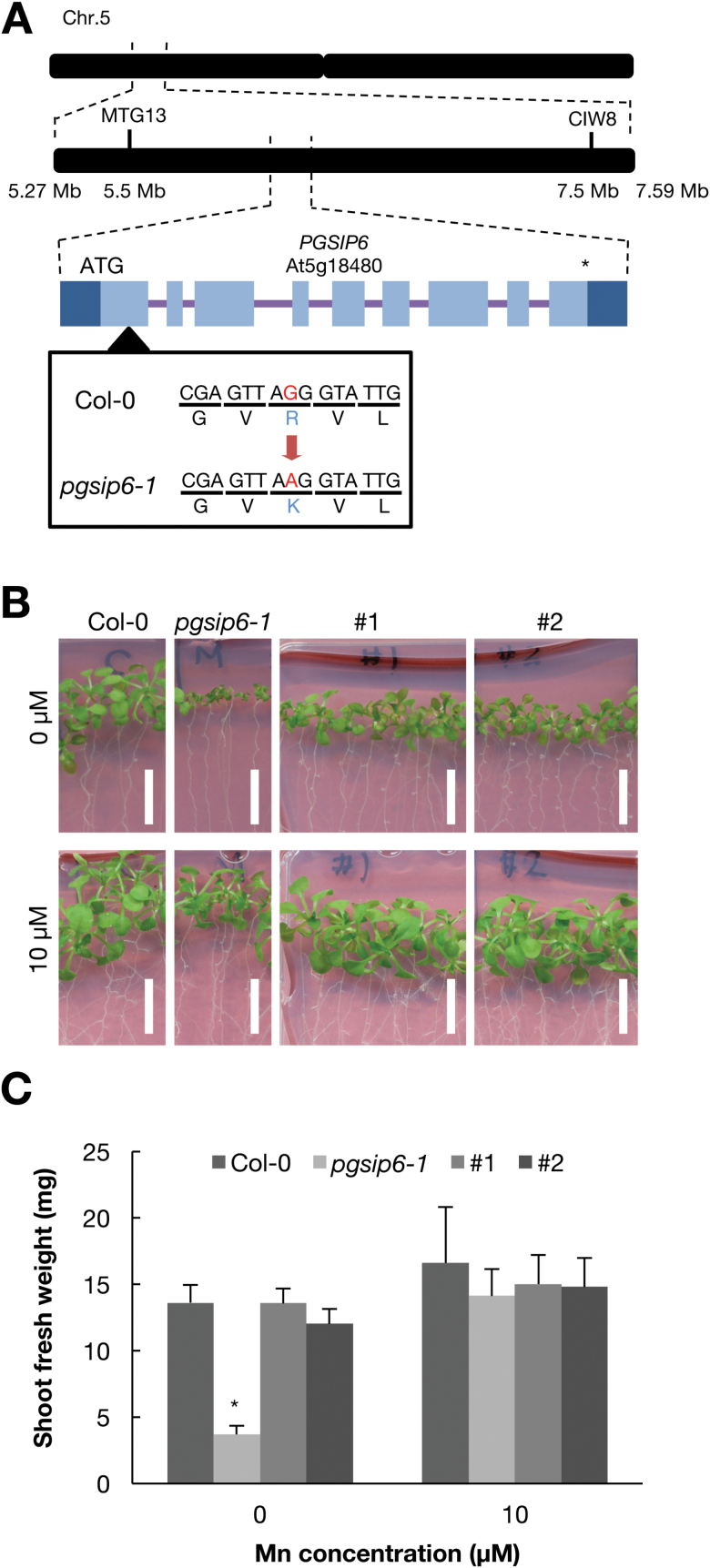
Disruption of PGSIP6 causes low-Mn sensitivity. (A) Genetic mapping and the mutation site of mutant 30-11. The markers and their positions are according to the TAIR10 database. Light blue boxes indicate exons encoding a protein, dark blue boxes are untranslated regions, and the bars between them are introns. MTG13 and GIW8 indicate the markers. (B) Complementation of *PGSIP6* with a construct in which genomic *PGSIP6* DNA with the promoter region was fused with GFP. Wild-type Col-0, *pgsip6-1*, and two complementation lines were grown under low-Mn and normal conditions for 14 d. Scale bars indicate 1 cm. (C) Shoot fresh weight of Col-0, *pgsip6-1*, and two complementation lines of *PGSIP6* (#1 and #2). Asterisks indicate significant differences between mutant 30-11 and the two complementation lines as determined by a Tukey–Kramer test (*P<0.05).

To identify the causal gene of 30-11, the mutant was crossed with L*er* and an F_2_ population was obtained. The F_2_ seeds were sown on low-Mn medium and the segregation ratio of the wild-type and the mutant was found to be 149:46. The result of a χ^2^ test (*P*=0.65) suggested that the low-Mn phenotype is caused by a single recessive mutation. From genetic mapping, the causal gene of 30-11 was found to be located between 5.27 and 7.59 Mb on chromosome 5, which contains 204 genes based on TAIR10 (Fig. 4A). To identify the causal gene, whole-genome sequencing of the mutant was performed using next-generation sequencer SOLiD™. There is only one non-synonymous mutation in this region, which is At5g18480, *Plant Glycogenin-like Starch Initiation Protein 6* (*PGSIP6*). In 30-11, Arg^47^ is substituted with a Lys. PGSIP6 is predicted to have a Mn^2+^-binding domain according to UniProt (http://www.uniprot.org; prediction based on Gibbons *et al.*, 2002). Hereafter, we refer to 30-11 as *pgsip6-1*. *PGSIP6* encodes inositol phosphorylceramide glucuronosyltransferase, which is involved in glycosyl inositol phosphorylceramide sphingolipid glycosylation (Tartaglio *et al.*, 2017).

To test whether PGSIP6 was the causal gene, we performed complementation analysis since a T-DNA line was not available. A genomic fragment of PGSIP6 fused with GFP (PGSIP6-GFP) was introduced into the mutant *pgsip6-1*. The phenotypic defect of *pgsip6-1* was rescued by PGSIP6-GFP (Fig. 4B, C). These results indicate that *PGSIP6* is the causal gene of *pgsip6-1*.

### Expression analysis of *PGSIP6*

The mRNA expression levels of *PGSIP6* in the shoots and roots of the wild-type under low- and normal Mn conditions were measured using real-time PCR. There were no significant differences either in the shoots or roots under low-Mn conditions compared to normal conditions (Supplementary Fig. S3).

### PGSIP6 is localized to the Golgi apparatus

**Fig. 5.**
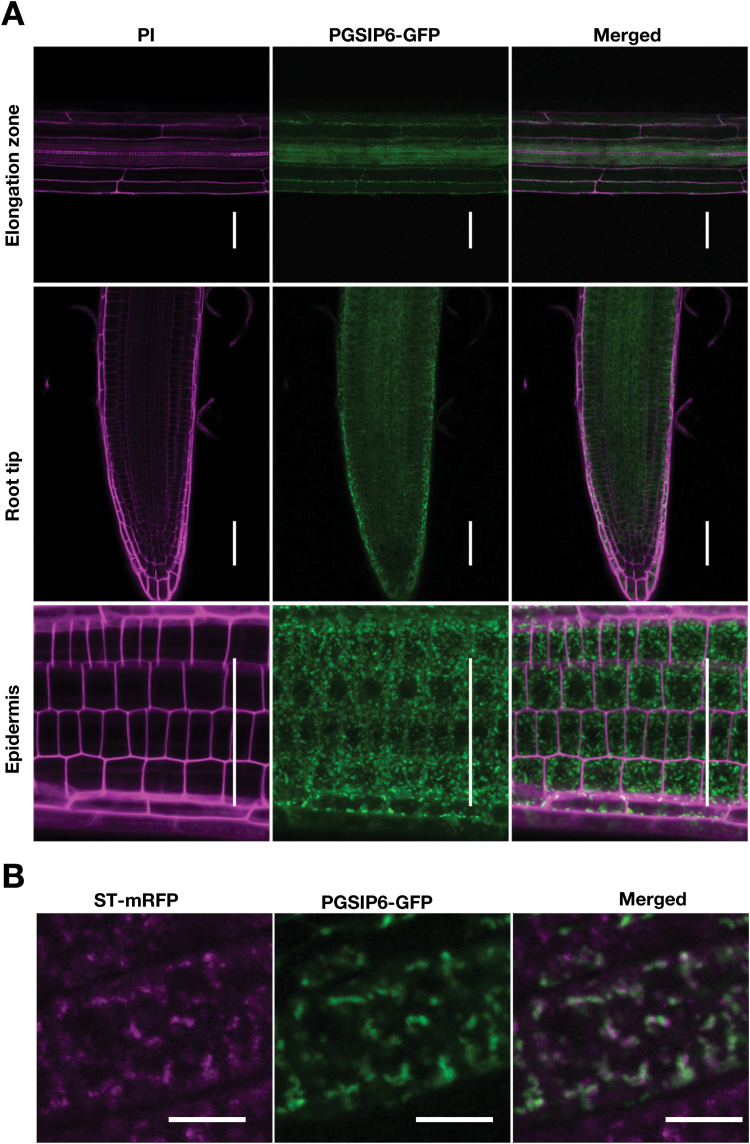
The expression and localization of PGSIP6-GFP in root cells. (A) Expression of the PGSIP6-GFP fusion protein in root tissues. Transformed plants expressing PGSIP6-GFP were observed using confocal microscopy. The cell wall was stained with propidium iodide (PI). Plants were grown on MGRL medium for 5 d. Scale bars indicate 50 μm. (B) Subcellular localization of the PGSIP6-GFP fusion protein in root cells. The fluorescence of PGSIP6-GFP was imaged with the Golgi marker ST-mRFP under normal Mn conditions. Golgi marker ST-mRFP fluorescence (left), GFP (middle), and merged images (right) are shown. Plants were grown on MGRL medium for 7 d and observed by confocal laser microscopy. Scale bars indicate 10 μm.

To identify tissue-specific expression and subcellular localization, we observed PGSIP6-GFP fluorescence of the complemented line (Fig. 4B). GFP fluorescence was widely observed in roots (Fig. 5A), being seen as dots, and it was partially co-localized with the trans-Golgi marker ST-mRFP (Ito *et al.*, 2012) (Fig. 5B), which is consistent with observations in a previous study after transient expression in tobacco epidermal cells (Rennie *et al.*, 2012; Nikolovski *et al.*, 2014). Some of the dots were not co-localized with ST-mRFP. Considering that ST-mRFP is localized to the trans-Golgi compartment, PGSIP6-GFP may be localized to the cis-Golgi as well.

## Discussion

In this study, we isolated eight low-Mn-sensitive mutants (Supplementary Fig. S1). We focused on two of them, *nramp1-3* and *pgsip6-1*, and identified two new alleles of *NRAMP1* and *PGSIP6* with altered low-Mn sensitivity, probably due to perturbation of the intracellular distribution and utilization of Mn, respectively.

### Effect of the *nramp1-3* mutation in NRAMP1 function

In this study, we found that *nramp1-3* has a mutation at an exon–intron junction that produces two different transcripts, either with the intron retained or with a 12-bp deletion (Fig. 3B). Two transcripts in *nramp1-3* could be produced, because one transcript with the intron led to a premature termination codon 51 bp downstream from the exon–intron junction, resulting in the addition of 17 amino acids (Supplementary Fig. S2B). The other transcript led to the loss of four amino acids in the second transmembrane domain, which is highly conserved among various plant species (Supplementary Figs S2B, S4). We confirmed that the phenotypes of *nramp1-3* were similar to the *nramp1-1* line. These data suggest that the low-Mn-sensitive phenotype of *nramp1-3* is due to the abnormal splicing of *NRAMP1*.

### Mn concentration-dependent phenotypes of *pgsip6-1*

*pgsip6-1* showed Mn-dependent phenotypes. One possibility is that mutated PGSIP6 has a low affinity for Mn^2+^ compared to the wild-type. PGSIP6 has a nucleotide-diphospho-sugar transferase domain (Campbell *et al.*, 1997) in its N-terminus and a transmembrane domain in its C-terminal region (Supplementary Fig. S5). *pgsip6-1* has a mutation in the N-terminal domain, where the Mn^2+^-binding site is predicted based on UniProt (http://www.uniprot.org). This prediction is based on the crystal structure of rabbit glycogenin (Gibbons *et al.*, 2002), in which three amino acid residues (Asp^101^, Asp^103^, and His^211^) directly bind with Mn^2+^. All of the amino acids are conserved in PGSIP6 (Asp^114^, Asp^116^, and His^248^) and also in other homologous genes (Supplementary Fig. S5). In addition, Rennie *et al.* (2012) showed that GUX1, the protein most similar to PGSIP6, requires Mn^2+^ for its activity. Therefore, PGSIP6 could require Mn^2+^ for its activity. The mutation site in *pgsip6-1* is Arg^47^, which is not close to the Mn^2+^-binding site. Therefore, *pgsip6-1* may result in a partial loss in PGSIP6 enzymatic activity, leading to the Mn^2+^-dependent phenotype. If the mutation caused more than a partial loss of PGSIP6 activity and reduced it to a large extent, then the alternative glucoronosylation pathway that is strongly dependent on Mn^2+^ would be exposed, resulting in the Mn^2+^-dependent phenotype.

Another possibility is that the product of PGSIP6 determines the Mn tolerance of plants. PGSIP6 is an inositol phosphorylceramide (IPC) glucuronosyltransferase (Rennie *et al.*, 2014) that produces glycosyl inositol phosphorylceramide (GIPC). Recent reports have indicated that GIPCs are a major component of plant plasma membranes and important for the maintenance of membrane microdomains (Markham *et al.*, 2006; Cacas *et al.*, 2016). However, to date there are no reports about the function of Mn^2+^ in relation to microdomain maintenance.

Until now, there has been no mutant allele available for *PGSIP6*. In the T-DNA heterozygous line, mutant pollen was found to be defective at later stages of reproduction (Rennie *et al.*, 2014; Tartaglio *et al.*, 2017). Our mutant could provide good material for functional analyses of PGSIP6 and its product, GIPC.

In conclusion, our study has shown that NRAMP1 and PGSIP6 are important for plant survival under low-Mn conditions. NRAMP1 functions as a Mn transporter and is involved in low-Mn tolerance. PGSIP6 is a Mn^2+^-binding glucuronosyltransferase that is associated with GIPC sphingolipid glycosylation. This study provides new insights to GIPC sphingolipid glycosylation, mediated by PGSIP6, and connects it to adaptation to low-Mn conditions.

## Supplementary data

Supplementary data are available at *JXB* online.

Fig. S1. Phenotypes of various low-Mn-sensitive mutants.

Fig. S2. Splicing variants of *nramp1-3*.

Fig. S3. Expression of *PGSIP6* under 0 and 10 µM Mn conditions.

Fig. S4. Multiple amino acid sequence alignment of NRAMPs.

Fig. S5. Multiple amino acid sequence alignment of PGSIPs.

Supplementary Figures S1-S5

## Abbreviations:

NRAMP: natural resistance-associated macrophage protein
GIPC: glycosyl inositol phosphorylceramide
PGSIP: plant glycogenin-like starch initiation protein.
