# Supplementary material for: Screening Arabidopsis thaliana mutants for low sensitivity to manganese identifies novel alleles of NRAMP1 and PGSIP6
Source: J Exp Bot. 2018 Jan 20;69(7):1795–803. doi: 10.1093/jxb/ery018 (PMC5888932; doi:10.1093/jxb/ery018)
Supplement: Supplementary Figures S1-S5 [file ery018_suppl_supplementary_figures_s1-s5.pdf]

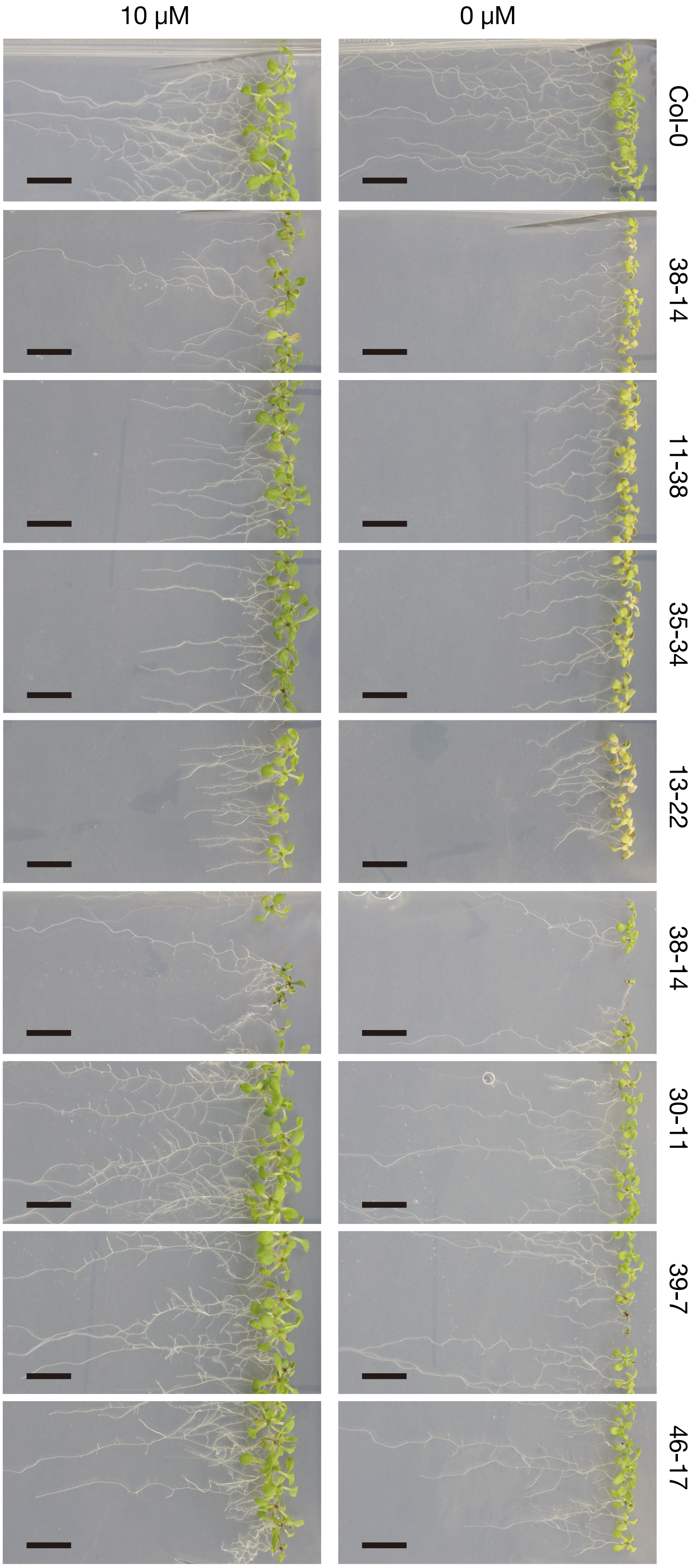

Fig. S1. Isolation of various mutants showed showing low-manganese Mn sensitivity. Mutant plants were grown in a MGRL medium at 22 °C for 15 days under low Mn (0 μM Mn) and normal Mn conditions (10 μM Mn). The scale bar in the photograph shows 1 cm.

Figure S1.

A

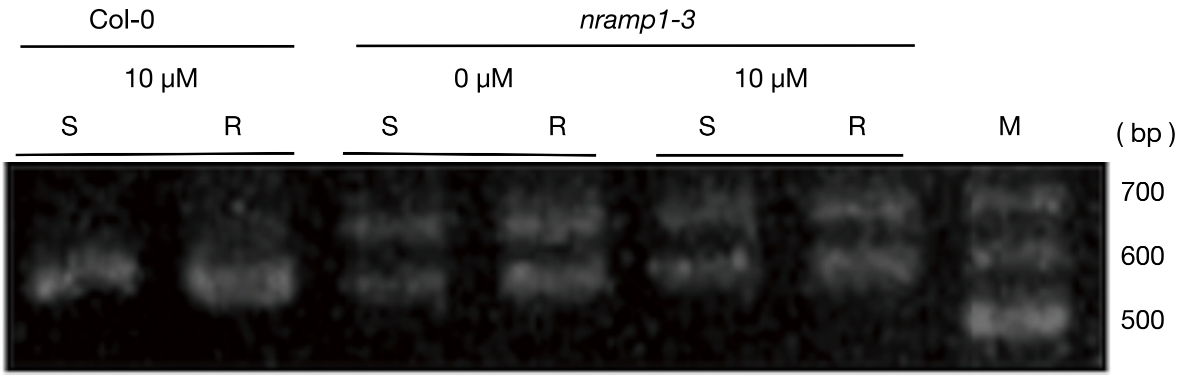

B

```
Col-0        1 MAATGSGRSQFISSSGGNRSFSNSPLIENSDSNQIIVSEKKSWKNFFAYLGPGFLVSIAY
nramp1-3a    1 MAATGSGRSQFISSSGGNRSFSNSPLIENSDSNQIIVSEKKSWKNFFAYLGPGFLVSIAY
nramp1-3b    1 MAATGSGRSQFISSSGGNRSFSNSPLIENSDSNQIIVSEKKSWKNFFAYLGPGFLVSIAY

Col-0       61 IDPGNFETDLQAGAHYKYELLWIILVASCAALVIQSLAANLGVVTGKHLAEQCRAEYSKV
nramp1-3a   61 IDPGNFETDLQAGAHYKYELLWIILVASCAALVIQSLAANLGVVTGDYHKNPLFSFSFMI
nramp1-3b   61 IDPGNFETDLQAGAHYKYELLWIILVASCAALVIQSLAANLG----KHLAEQCRAEYSKV

Col-0      121 PNFMLWVVAEIAVVACDIPEVIGTAFALNMLFSIPVWIGVLLTGLSTLILLALQKYGVRK
nramp1-3a  121 QVY*--------------------------------------------------------
nramp1-3b  117 PNFMLWVVAEIAVVACDIPEVIGTAFALNMLFSIPVWIGVLLTGLSTLILLALQKYGVRK

Col-0      181 LEFLIAFLVFTIAICFFV
nramp1-3a      ------------------
nramp1-3b  177 LEFLIAFLVFTIAICFFV
```

Fig. S2. The point mutation in the splicing junction between the *NRAMP1* fourth intron and fourth exon of *nramp1-3* results in two different proteins.

(A) Analysis of gene *NRAMP1* expression of in wild type and *nramp1-3* under normal Mn normal and Mn deficiency conditions by RT-PCR, using specific primers for the gene *NRAMP1*. Mutation in the *NRAMP1* intron splicing site results in two variant forms. S: shoot, R: root, and M: marker.

(B) Amino acid sequence alignment near the mutation site in Col-0 and the two types of *NRAMP1* transcript isoforms in *nramp1-3*. “nramp1-3a“ ” and “nramp1-3b” represent the amino acid sequence corresponding to the intron intron-inserted transcript and the exon transcript which has a 12 12-bp deletion, respectively, in panel A and also corresponding to Fig. 3B.

Figure S2.

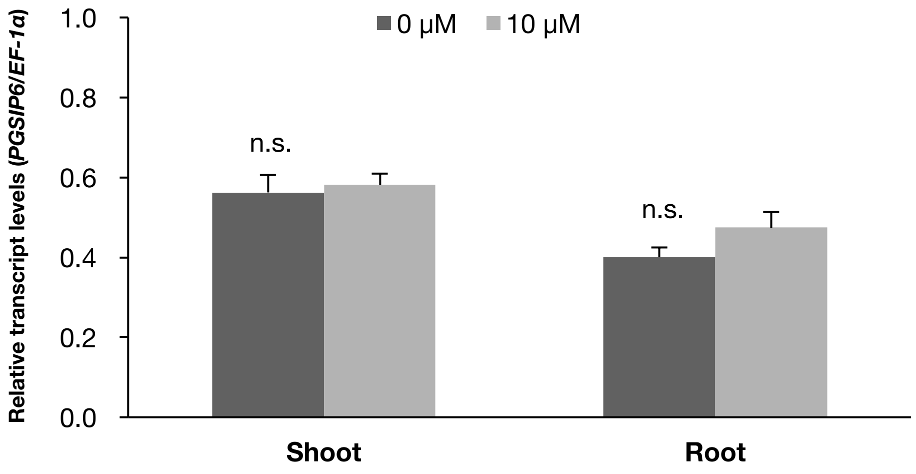

Fig. S3. The *PGSIP6* expression of Col-0 in shoots and roots under normal Mn normal and Mn deficiency conditions. Plants of *pgsip6-1* were grown under 0 μM Mn (deficiency), and 10 μM Mn (normal) conditions for 14 days on vertically vertically-placed MGRL mediumplates. The expression analysis was determined using Real real-Time time RT-PCR. Levels of *PGSIP6* mRNA were normalized to those of *EF-1α* in the same samples. Bars represent the mean ± standard deviation, Nn = 5. The "n.s." indicates no significant difference was detected for theof expression between normal Mn normal and Mn deficiency conditions by Student's t-test.

Figure S3.

```
Arabidopsis thaliana     1 MAATGSGRSQFISSSGGNRSFSNSPLIENSDSNQIIVSEKKSWKNFFAYLGPGFLVSIAY
Zea mays                 1 -------MDAAQVPAAVVVDVGALTGPIG--SEAPAAPKGPAWKRFLYHVGPGFMVCLAY
Sorghum bicolor          1 ------MDAGAHVPAGVVVDIEALAGPAVGSEVPAAPPKGPAWKRFLYHVGPGFMVCLAY
Oryza sativa             1 ------------MGVTKAEAVAATGKVVDDIEALADLRKEPAWKKFLSHIGPGFMVCLAY
Brassica rapa            1 MAASASERPQFISSNGGNRSFSNAPLIDNSDPNQIIVPEKKSWKNFFAYLGPGFLVLIAY

Arabidopsis thaliana    61 IDPGNFETDLQAGAHYKYELLWIILVASCAALVIQSLAANLGVVTGKHLAEQCRAEYSKV
Zea mays                52 LDPGNLGTDLQAGADHRYELLWVTLIGLIFALIIQSLSANLGVVTGRHLAELCKTEYPAW
Sorghum bicolor         55 LDPGNLGTDLQAGADHRYELLWVTLIGLIFALIIQSLSANLGVVTGRHLAELCKTEYPAW
Oryza sativa            49 LDPGNMETDLQAGANHKYELLWVILIGLIFALIIQSLSANLGVVTGRHLAELCKTEYPVW
Brassica rapa           61 IDPGNFETDLQAGAQYKYELLWIILVASCAALVIQSLAANLGVVTGKHLAEHCRAEYSKV

Arabidopsis thaliana   121 PNFMLWVVAEIAVVACDIPEVIGTAFALNMLFSIPVWIGVLLTGLSTLILLALQKYGVRK
Zea mays               112 VRICLWLLAELAVIAADIPEVIGTAFAFNILFHVPLWVGVLITGSSTLLLLGLQKYGVRK
Sorghum bicolor        115 VRICLWLLAELAVIAADIPEVIGTAFAFNLLFHIPLWVGVLITGSSTLLLLGMQKYGVRK
Oryza sativa           109 VKTCLWLLAELAVIASDIPEVIGTGFAFNLLFHIPVWTGVLIAGSSTLLLLGLQRYGVRK
Brassica rapa          121 PNFLLWVVAEIAVVACDIPEVIGTAFALNMLFSIPVWIGVLLTGLSTLMLLALQQYGVRK

Arabidopsis thaliana   181 LEFLIAFLVFTIAICFFVELHYSKPDPGEVLHGLFVPQLKGNGATGLAISLLGAMVMPHN
Zea mays               172 LELLVGLLVFVMAACFFIEMSIVKPPAEEVIHGLFIPSLSGPGATGDTIALLGALVMPHN
Sorghum bicolor        175 LELLVGLLVFVMAACFFIEMSIVKPPAKEVIYGLFVPSLSGSGATGDTIALLGALVMPHN
Oryza sativa           169 LEVVVALLVFVMAGCFFVEMSIVKPPVNEVLQGLFIPRLSGPGATGDSIALLGALVMPHN
Brassica rapa          181 LEFLIAFLVFTIALCFVIELHYSKPDPGEVLHGLFVPQLKGNGATGLAISLLGAMVMPHN

Arabidopsis thaliana   241 LFLHSALVLSRKIPRSASGIKEACRFYLIESGLALMVAFLINVSVISVSGAVCNAPNLSP
Zea mays               232 LFLHSALVLSRNTPSSVRGINDACRFFLLESGVAMFVALIINICIISVSGTVCNSSNVSP
Sorghum bicolor        235 LFLHSALVLSRNTPSSVRGIKDACRFFLLESGVAMFVALLINICIISVSGTVCNSSNLSS
Oryza sativa           229 LFLHSALVLSRNTPASAKGMKDACRFFLFESGIALFVALLVNIAIISVSGTVCNATNLSP
Brassica rapa          241 LFLHSALVLSRKIPRSSTGIKEACRFYLIESGLALMIAFLINVSVISVSGAVCNAPDLTP

Arabidopsis thaliana   301 EDRANCEDLDLNKASFLLRNVVGKWSSKLFAIALLASGQSSTITGTYAGQYVMQG-----
Zea mays               292 DDSAKCSDITLDSSSFLLRNVLGNSSAVVYGVALLACGISSSITGTYAGQYIMQIVILEA
Sorghum bicolor        295 VDSAKCSDITLDSSSFLLRNVLGNNSAVVYGVALLACGISSSITGTYAGQYIMQG-----
Oryza sativa           289 EDAVKCSDLTLDSSSFLLRNVLGKSSATVYGVALLASGQSSTITGTYAGQYVMQG-----
Brassica rapa          301 EDRAKCEDLDLNKASFLLRNVVGKWSSKLFAIALLASGQSSTITGTYAGQYVMQG-----

Arabidopsis thaliana   356 -----------FLDLRLEPWLRNLLTRCLAIIPSLIVALIGGSAGAGKLIIIASMILSFE
Zea mays               352 RFACVWLLAQGFLDIKMRQWLRNLMTRSIAIIPSLIVAIIGGSSGAGRLVIIASMILSFE
Sorghum bicolor        350 -----------FLDIKMKQWLRNLMTRSIAIVPSLIVAIIGGSSGAGRLIIIASMILSFE
Oryza sativa           344 -----------FLDIKMKQWLRNLMTRSIAIVPSLIVSIIGGSSGAGRLIVIASMILSFE
Brassica rapa          356 -----------FLDLRLEPWLRNFLTRCLAIIPSLIVALIGGSAGAGKLIIIASMILSFE

Arabidopsis thaliana   405 LPFALVPLLKFTSCKTKMGSHVNPMAITALTWVIGGLIMGINIYYLVSSFIKLLIHSHMK
Zea mays               412 LPFALIPLLKFSSSSSTMGQHKNSIFIIGFSWILGFVIIGINIYFLSSKFVGWILHNSLQ
Sorghum bicolor        399 LPFALIPLLKFSSSSNTMGQHKNSIYIIGFSWILGSVIIGINIYFLCSKFVGWILHNSLP
Oryza sativa           393 LPFALIPLLKFSSSSNKMGENKNSIYIVGFSWVLGFVIIGINIYFLSTKLVGWILHNALP
Brassica rapa          405 LPFALVPLLKFTSCKTKMGSHVNHMAITTLTWVIGALIMGINIYYIISSFTKLLIHSHMK

Arabidopsis thaliana   465 LILVVFCGILGFAGIALYLAAIAYLVFRKNRVATSLLISRDSQNVETLPRQDIVNMQLPC
Zea mays               472 IYANILIGIVVFPLMLIYVCAVIYLTLRKETIKFVACGELQTIETDKSKLANGSNMEEKK
Sorghum bicolor        459 IYANILIGIIVFPLMLLYICAVIYLTLRKETIKFVPCGELQAIETDKSKVADDSNNEEKE
Oryza sativa           453 TFANVLIGIVLFPLMLLYVVAVIYLTFRKDTVKFVSRRELQAGDDTEKAQVATCVADEDS
Brassica rapa          465 LALVIFCGILGFSGIAIYLASIAYLVFRKNRKASPLLASTNSQTVETLPRQDIVDMQLHG

Arabidopsis thaliana   525 RVSTSDVD
Zea mays               532 DQLV----
Sorghum bicolor        519 YVSYNGI-
Oryza sativa           513 KEPPV---
Brassica rapa          525 KAAASDLD
```

Figure S4.

Fig. S4. Multiple amino acid sequences alignment in of NRAMP1 (AT1g80830) and homologs using ClustalW. Amino acid sequences of NRAMP1 and homologs from *Arabidopsis thaliana* (At1g80830), *Zea mays* (Zm00001d005479), *Sorghum bicolor* (SORBI_3002G095600), *Oryza sativa* (OSNPB_070258400), and *Brassics Brassica rapa* (Bra035149). The red outlined residues indicate the 12-bp exon deleted deletion variant found in *nramp1-3*. Blue lines indicate the predicted transmembrane domains (Curie *et al*., 2000; Haines *et al*., 2015).

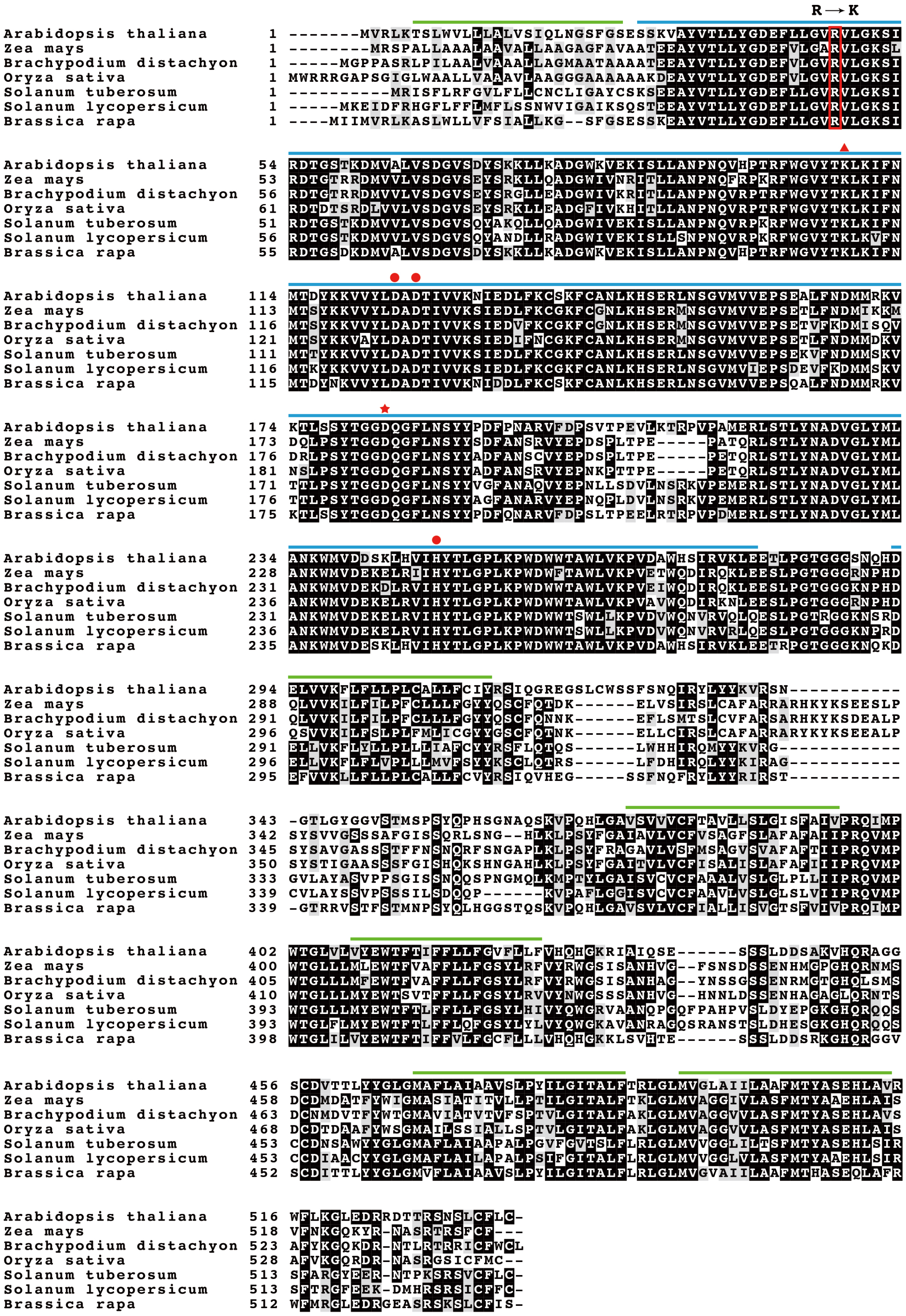

Figure S5.

Fig. S5. Multiple sequences alignment in of PGSIP6 (AT5g18480) and homologs using ClustalW. The Aamino acid sequences of PGSIP6 and homologs from *Zea mays* (Zm2G166903), *Brachypodium distachyon* (Bd3g49197), *Oryza sativa* (Os02g41520), *Solanum tuberosum* (St20316), *Solanum lycopersicum* (Sl05g055040), and *Brassica rapa* (Br006462). Blue lines indicate the nucleotide-diphospho-sugar transferases domain (Uniprot: http://www.uniprot.org; Gibbons *et al*., 2002). Green lines indicate the predicted transmembrane domains. The Rred circles, triangle, and star indicate the predicted manganese binding site, the active site, and the catalytic active site, respectively (UniProt: http://www.uniprot.org; Gibbons *et al*., 2002).
